# Integrated virtual screening and *in vitro* studies for exploring the mechanism of triterpenoids in Chebulae Fructus alleviating mesaconitine-induced cardiotoxicity via TRPV1 channel

**DOI:** 10.3389/fphar.2024.1367682

**Published:** 2024-03-04

**Authors:** Liangliang Song, Shuo Mi, Ying Zhao, Ziqin Liu, Jing Wang, Hongyue Wang, Wenhui Li, Jiasheng Wang, Wenting Zu, Hong Du

**Affiliations:** School of Chinese Materia Medica, Beijing University of Chinese Medicine, Beijing, China

**Keywords:** Chebulae Fructus triterpenoids, mesaconitine, TRPV1, cardiotoxicity, pharmacophore model, molecular docking, 3D-QSAR model

## Abstract

**Background:** In traditional Mongolian or Tibetan medicine in China, Chebulae Fructus (CF) is widely used to process or combine with aconitums to decrease the severe toxicity of aconitums. Researches in this area have predominantly focused on tannins, with few research on other major CF components for cardiotoxicity mitigation. The present study aimed to clarify whether triterpenoids can attenuate the cardiotoxicity caused by mesaconitine (MA) and investigate the mechanism of cardiotoxicity attenuation.

**Methods:** Firstly, the pharmacophore model, molecular docking, and 3D-QSAR model were used to explore the mechanism of CF components in reducing the toxicity of MA mediated by the TRPV1 channel. Then three triterpenoids were selected to verify whether the triterpenoids had the effect of lowering the cardiotoxicity of MA using H9c2 cells combined with MTT, Hoechst 33258, and JC-1. Finally, Western blot, Fluo-3AM, and MTT assays combined with capsazepine were used to verify whether the triterpenoids reduced H9c2 cardiomyocyte toxicity induced by MA was related to the TRPV1 channel.

**Results:** Seven triterpenoids in CF have the potential to activate the TRPV1 channel. And they exhibited greater affinity for TRPV1 compared to other compounds and MA. However, their activity was relatively lower than that of MA. Cell experiments revealed that MA significantly reduced H9c2 cell viability, resulting in diminished mitochondrial membrane potential and nuclear pyknosis and damage. In contrast, the triterpenoids could improve the survival rate significantly and counteract the damage of MA to the cells. We found that MA, arjungenin (AR), and maslinic acid (MSA) except corosolic acid (CRA) upregulated the expression of TRPV1 protein. MA induced a significant influx of calcium, whereas all three triterpenoids alleviated this trend. Blocking the TRPV1 channel with capsazepine only increased the cell viability that had been simultaneously treated with MA, and AR, or MSA. However, there was no significant difference in the CRA groups treated with or without capsazepine.

**Conclusion:** The triterpenoids in CF can reduce the cardiotoxicity caused by MA. The MSA and AR function as TRPV1 agonists with comparatively reduced activity but a greater capacity to bind to TRPV1 receptors, thus antagonizing the excessive activation of TRPV1 by MA.

## 1 Introduction

According to traditional Mongolian medical theory, Chebulae Fructus (the fruits of *Terminalia chebula* Retz. or *Terminalia chebula* Retz, var. *Tomentella* Kurt., named Hezi in Chinese, CF) has a detoxifying function and may not only treat a variety of toxic diseases but also reconcile the drug properties and lessen the toxicity of poisonous medications (Nigam et al., 2020; Zhou et al., 2022; Ai et al., 2023). According to the records in the Tibetan medical classic “Jingzhu Materia Medica,” CF has a therapeutic effect on various ailments and is the king of all remedies (Dimar, 1986). It can be inferred from the phenomenon that among the commonly used 200 prescriptions in the Ministry standards of Tibetan medicine, 125 prescriptions all contain the CF (Song et al., 2023). In the use habits of ethnic minorities, it is more commonly used together with the aconitum herbs, such as *Aconitum pendulum* Busch, *Aconiti kusnezoffii* radix. In order to reduce the toxicity or preservation effect, CF decoction processing aconitums or compatibility aconitums with CF are popular methods in clinical practice (Li et al., 2019; Li et al., 2021), and they are often used in the form of pills or powders, such as Naru Sanwei Wan (Guo et al., 2023). However, the mechanism behind the attenuation toxicity remains unclear and requires further exploration.

Diester diterpenoid alkaloids (DDAs), represented by mesaconitine (C_33_H_45_NO_11_, CAS: 2752-64-9, MA), are the main toxic components of aconitums (Wei et al., 2019; Chen et al., 2023). The toxicity of MA is quite potent; the median lethal dose (LD_50_) of oral administration, subcutaneous injection, intraperitoneal injection, and intravenous injection in mice is 1.9 mg/kg, 0.20 mg/kg ∼ 0.38 mg/kg, 0.20 mg/kg ∼ 0.30 mg/kg, 0.068 mg/kg ∼ 0.13 mg/kg, respectively (Liu et al., 2016b; Yang et al., 2018; Chan et al., 2021). Cardiotoxicity is the main manifestation of aconitum herbs toxicity. It can lead to abnormal electrocardiogram changes, palpitations and arrhythmia, especially premature ventricular beats, which are the most common (Feng, 2019; Ye et al., 2021; Chen et al., 2023). For cells, it can lead to myocardial cell apoptosis, which is manifested in reducing the survival rate of rat H9c2 cardiomyocytes, causing cell nuclear condensation, damage and an increase in mitochondrial membrane potential (Li et al., 2020; Han et al., 2022c). There are multiple mechanisms of cardiotoxicity, including ion channels, protein expression, oxidative stress, energy metabolism damage, and so on (Sun et al., 2014; Zhou et al., 2021; Gao et al., 2022). Among them, ion channels are an important way to exert the efficacy and toxicity of diester alkaloids. When the function of ion channels is aberrant, the proportion of each channel in the myocardium will be destroyed, which will induce cardiac diseases such as arrhythmia (Fu et al., 2007; Yang et al., 2021).

The transient receptor potential vanilloid type 1 channel (TRPV1) is widely distributed in the heart (Peng and Li, 2010). It is a non-selective ion channel that dominates the influx of calcium ions. Activating the TRPV1 channel results in an influx of extracellular calcium and the release of intracellular calcium, causing an increase in intracellular calcium concentration (Sappington et al., 2009). Since calcium serves as the second messenger in many intracellular signaling pathways, TRPV1 can thus participate in various physiological processes of cells, including energy metabolism, cell proliferation, and apoptosis (Wu et al., 2015; Zhai et al., 2020). Researchers have discovered that TRPV1 channels are present in rat H9c2 cardiomyocytes and can be triggered in response to hypoxia/reoxygenation stimulation. The TRPV1 agonist capsaicin can dramatically worsen apoptosis, raise intracellular Ca^2+^ levels, increase mitochondrial superoxide release, and then lower mitochondrial membrane potential when mixed with it. However, the TRPV1 antagonist capsazepine can reverse this effect because it is a competitive small-molecule TRPV1 inhibitor and can block the activation of the TRPV1 channels by capsaicin or other agonists. Overactivation of TRPV1 channels in the heart induces apoptosis of cardiomyocytes, facilitating their death and contributing to ventricular tachycardia and fibrillation in individuals with myocardial infarction (Sun et al., 2014). This is a significant mechanism of cardiotoxicity and a notable pathway of cardiotoxicity as well. The TRPV1 channel is a significant pathway of cardiotoxicity induced by DDAs. The previous study found that the DDAs in aconitums caused intracellular calcium overload due to excessive activation of the TRPV1 channel in H9c2 cardiomyocytes. This led to damage to various organelles, resulting in a significant amount of apoptosis, which is one of the ways aconitum herbs produce cardiotoxicity (Han et al., 2022b; Han et al., 2022c).

CF is rich in tannins such as gallic acid and chebulic acid, as well as triterpenoids such as arjunolic acid, maslinic acid (MSA), corosolic acid (CRA), and chebulatriol. It also contains flavonoids, amino acids, and volatile compounds (Li et al., 2022). The components in CF might be involved in lowering toxicity, regardless of whether CF is compatibile with aconitum herbs or used as an excipient to process aconitums (Liu et al., 2016a; Zhi et al., 2020a). Academics have conducted studies and found that the chemical constituents interactions between aconitums and the auxiliary substance CF can lower the cardiotoxicity of aconitums (Liu et al., 2013). Network pharmacology was employed to investigate how CF reduces the cardiac toxicity of aconitums. A total of 15 components in CF were screened, including seven tannins, five triterpenoids, and three alkaloids. They reduce cardiotoxicity by involving numerous protein functions, signaling pathways, and biological processes (Li et al., 2018). Our research group has verified that tannins and phenolic acids from CF can significantly reduce the mitochondrial membrane potential elevation and intracellular Ca^2+^ overload caused by aconitine and MA in rat H9c2 cardiomyocytes through the TRPV1 channel, thereby improving mitochondrial function and inhibiting the cardiotoxicity of aconitine (Han et al., 2020; Han et al., 2022b; Han et al., 2022c). When we were committed to the study of tannins, we accidentally found that triterpenoids in CF, while not as abundant as tannins, have a significant impact on antioxidant and cardioprotective properties. These effects were achieved through antioxidation, inhibition of intracellular calcium overload in cardiomyocytes, and prevention of oxidative stress damage in cardiomyocytes (Sumitra et al., 2001; Zou et al., 2017; Wang, 2019; An et al., 2020). Coincidentally, this aligns with the mechanism by which DDAs in aconitums exert cardiotoxicity. Furthermore, research has shown that the triterpenoids in CF have the potential to activate TRPV1, which may play a role in attenuating cardiotoxicity (Mi et al., 2023). Consequently, further research is vital to identify whether the triterpenoids present in CF possess the ability to alleviate the cardiotoxicity of MA and if the attenuation mechanism involves the TRPV1 channel.

In the present study, the pharmacophore model based on the common feature structure of ligands (TRPV1 agonists) was used to search for drug molecules that are identical or similar to the pharmacophore model screened in the database (Acharya et al., 2011; Qiao and Zhang, 2014). Then the binding ability of biological macromolecules and small molecules was evaluated according to the scoring function of molecular docking, and the molecules with reasonable binding modes and high prediction scores were selected (Yang, 2010). Subsequently, the 3D-QSAR model was used to investigate the structure-activity relationship and perform activity prediction afterward (Hansch et al., 1962), thereby screening out the components of CF that attenuate the cardiotoxicity of MA through the TRPV1 channel. The attenuation effect was then verified by cell experiments, and the chosen attenuation concentration was used to further verify whether the effect was related to the TRPV1 channel. This provided experimental support for the theoretical underpinnings and molecular mechanism of CF in reducing cardiotoxicity induced by aconitums.

## 2 Materials and methods

### 2.1 Virtual screening based on pharmacophore model

#### 2.1.1 Preparation of ligands

The pharmacophore model is particularly useful when the receptor structure is unknown or the mechanism of action is unclear. Based on the common features of a series of small molecule ligands, the pharmacophore model is generated, and then the compound database is searched for active compounds. Pharmacophore model has become an important tool in drug discovery, particularly for fast and effective screening of new bioactive molecules.

By searching the small molecule database PubChem (https://pubchem.ncbi.nlm.nih.gov/), TCMSP (http://tcmspw.com/tcmsp.php), TCMID (http://www.Megabionet.org/tcmid/) and relevant literature, we found 60 components in CF (Supplementary Table S1). And then, we searched for 10 TRPV1 agonists and 10 TRPV1 antagonists from the literature (Supplementary Table S2) and downloaded small molecule ligands (Gavva, 2008; Gunthorpe and Szallasi, 2008; Burgess and Williams, 2010; Li et al., 2011; Szolcsányi and Sándor, 2012; Sugimoto et al., 2013; Yin, 2019; Choi et al., 2020; Duarte et al., 2020; Mazeto et al., 2020; More et al., 2020; Nie, 2020). We also had to randomly download 90 compounds from the PubChem database that did not have well-defined TRPV1 agonist activity for later verification. All small molecular structures were reconstructed with energy minimization in the MMFF94 forcefield through Chem 3D (version 15.1.0) software. The obtained conformations were used for subsequent analysis.

#### 2.1.2 Generation of pharmacophore model

**FIGURE 1:**
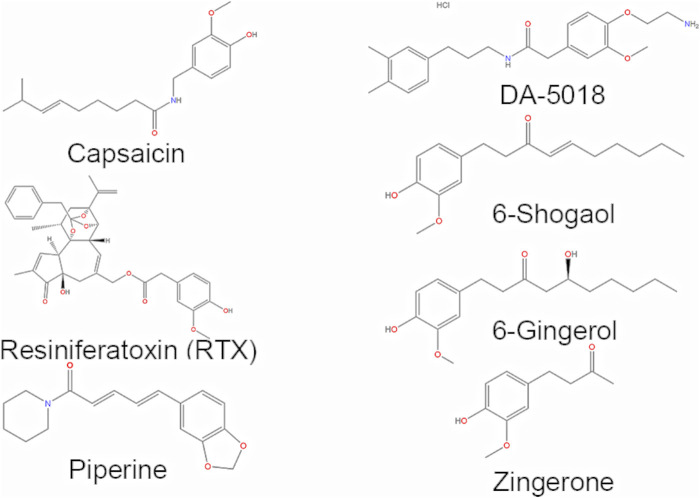
Structures of the TRPV1 agonists selected as the training set.

Firstly, seven representative components were selected from the downloaded components with TRPV1 agonists to form a training set (Figure 1), including piperine, capsaicin, 6-gingerol, 6-shogaol, zingerone, resiniferatoxin (RTX), and DA-5018. And then the HipHop approach of the Common Feature Pharmacophore Generation module in Discovery Studio (version 4.0) was used to automatically generate the pharmacophore models based on the common structural features of the small molecules in the training set. The Principal value of the active compounds was set to 2, indicating that the small molecules are active, and the MaxOmitFeat value was set to 1, indicating that the number of characteristic elements in each molecule that allow not match to the pharmacophore model is 1. Then the Hydrophobe, Donor, Acceptor, Ionizable Positive and Ring Aromatic were selected as the characteristic elements of pharmacophore in the HipHop module. The best mode was adopted for superposition, and the Maximum Conformation was set to 255. Only the model whose energy difference with the lowest conformation was less than 20 kcal/mol was saved. Eventually, we would get the 10 pharmacophore models with the higher score.

#### 2.1.3 Pharmacophore model validation

Using the Ligand Profiler module in Discovery Studio, the 10 generated pharmacophores were evaluated. The molecules in the test set consisted of 10 TRPV1 agonists (Supplementary Table S2) and 90 components with no reported TRPV1 agonist activity. According to the Rank value and the matching degree of the test set molecules, one pharmacophore was selected for subsequent screening, and the final selected pharmacophore was characterized by a high score, a high matching degree with the active ingredient, and a low matching degree with the inactive ingredient.

#### 2.1.4 Virtual screening

The Search 3D Database module in Discovery Studio was used to screen components with potentially activated TRPV1 channel activity based on the generated pharmacophore model.

### 2.2 Virtual screening based on molecular docking

#### 2.2.1 Preparations for molecular docking

Molecular docking is a method used to study the interaction between small molecules and target proteins. It can efficiently and accurately predict drug activity and selectivity, thus avoiding blind screening and reducing the cost of screening active compounds.

In the molecular docking experiment, the structure of MA also needed to be downloaded from PubChem (https://pubchem.ncbi.nlm.nih.gov/). We still use the small molecule ligands in Supplementary Table S1. Then we handled them with energy minimization and picked root and rotatable keys via Autodock Vina software (version 1.5.6), which was also used for the subsequent molecular docking calculations.

The two TRPV1 protein receptors that we selected for molecular docking were downloaded from the Protein Data Bank (PDB) database (http://www.ncbi.nlm.nih.gow/protein/). They are respectively the structure of TRPV1 in complex with DkTx and RTX (PDB ID code: 5IRX; Resolution: 2.95Å), and the structure of TRPV1 in complex with capsazepine (PDB ID code: 5IS0; Resolution: 3.43Å) (Gao et al., 2016).

To conduct virtual screening, firstly the active binding sites of molecular targets were determined by Discovery Studio. RTX is a TRPV1 agonist that interacts with residues: ARG557, LEU553, ALA566, ASN551, THR550, ILE569, MET547, ALA546, PHE591, LEU669, ALA665, TYR511, PHE543, LEU515, PHE507, ILE514, VAL518 of 5IRX (Supplementary Figures S1A, B). Capsazepine is a TRPV1 antagonist which interacts with residues: GLU570, LEU553, TYR554, TYR511, SER512, THR550, MET547, ALA665, PHE543, LEU662 of 5IS0 (Supplementary Figures S1C, D). The comparison shows that the TRPV1 agonist and the TRPV1 antagonist act on the same region of the TRPV1 protein, while the residues that act are not the same.

We needed to pretreat the protein receptors, including dehydration, hydrogenation, *etc.* The active sites of proteins also needs to be determined. What we obtained from the PDB database were the protein-ligand complexes. By analyzing the spatial position relationship between the receptor and the original ligand at the docking site, the information of the docking site can be obtained (Feinstein and Brylinski, 2015). The coordinates of RTX in 5IRX were (X = 93.69, Y = 114.04, Z = 129.88), and generated a square docking box with a side length of 21.275 centered on this site. The coordinates of capsazepine in 5IS0 were (X = 108.115, Y = 94.986, Z = 102.379), and generated a square docking box with a side length of 21.141 centered on this site. In addition, we researched the necessary literature to corroborate the location (Li and Yang, 2018).

#### 2.2.2 Molecular docking

Molecular docking was carried out on Autodock Vina. During the procedure, the semi-flexible docking method was used, that is, the protein receptor was rigid and the partial bond of the small molecules ligand could rotate. The TRPV1 agonists were placed into the indicated cave of the 5IRX receptor, and the TRPV1 antagonists were placed in the specified box in the 5IRX receptor. And then the components of CF (Supplementary Table S1) and the MA were docked to the two receptors severally and evaluated their binding ability.

### 2.3 Virtual screening based on 3D-QSAR model

#### 2.3.1 Construction of 3D-QSAR model

The 3D-QSAR model is a pharmacophore model with activity prediction ability based on a series of compounds with clear activity values for specific biological targets. The model was used to predict the related activities of new compounds that were not experimentally determined or even not synthesized. It is highly efficient, especially in the screening of compounds in large databases has an irreplaceable advantage.

Twelve TRPV1 agonists with the same EC_50_ data source were selected from the BindingDB database (https://www.bindingdb.org/). The corresponding small molecule ligands were downloaded from the PubChem database (https://pubchem.ncbi.nlm.nih.gov/) and the pEC_50_ data were added to the small molecule ligands (Supplementary Table S3). Chem 3D software was used to minimize the MMFF94 force field energy of all small molecule ligands. The first seven small molecules in the table were selected to form the training set, while all 12 TRPV1 agonists in Supplementary Table S3 will be used as the test set.

Using the Create 3D QSAR Model module in Discovery Studio, a 3D-QSAR model with activity prediction ability was constructed based on the common structural features and pEC_50_ values of small molecules selected in the training set, and the constructed model was verified by the test set. Representative components and a 3D-QSAR model were selected to generate visual contour maps of the electrostatic field coefficient and stereo field coefficient.

#### 2.3.2 Prediction of activity based on 3D-QSAR model

The Calculate Molecular Properties module in Discovery Studio was used to predict the activity of prepared triterpenoids and MA using the already constructed 3D-QSAR model, and output the results.

### 2.4 Verification of triterpenoids alleviating the H9c2 cardiomyocyte toxicity caused by MA

#### 2.4.1 Preparations of materials and cell culture

Rat cardiomyocyte H9c2 cells were purchased from the Cell Resource Center, IBMS, CAMS/PUMC (CRC/PUMC, Beijing, China). The H9c2 cells were cultured in high-glucose dulbecco’s modified eagle medium (DMEM) medium (VivaCell Biosciences, China) supplemented with 10% fetal bovine serum (FBS) (VivaCell Biosciences, China) and 1% penicillin-streptomycin (Lablead, China), and then incubated at 37°C with 5% CO_2_.

Corosolic acid (CRA, >98%, DSTDX000501) Maslinic acid (MSA, >98%, DST220526-039) Arjungenin (AR, >98%, DST210106-442) Mesaconitine (MA, >98%, DSTDX002501) were purchased from DeSiTe (Chengdu, China). Capsazepine (>98%, D15O9F2190) was obtained from Yuanye Biochemical (Shanghai, China). All test samples were accurately weighed, and dissolved by dimethyl sulfoxide (DMSO) (Sigama-Aldrich, United States) to obtain concentrated solutions, and then preserved at 4°C. They were diluted to the corresponding concentration with medium prior to use and allowed the DMSO concentration less than 0.5%.

#### 2.4.2 Cell viability of triterpenoids on H9c2 cardiomyocyte

Cell viability was assessed using the 3-(4,5-Dimethylthiazol-2-yl)-2,5-diphenyltetrazolium bromide (MTT) (Lablead, Beijing, China) assay. Initially, the MTT assay was utilized to identify a safe and non-toxic concentration range of triterpenoids to prevent the interference of triterpenoids with the survival rate of H9c2 cells and the subsequent experimental results. H9c2 cells were cultured with a medium containing various concentrations of triterpenoids for 24 h. The following grouping and dosages were used: 1) control group: H9c2 cells were cultured without any treatment; 2) CRA group: H9c2 cells were treated with different concentrations of CRA (8, 16, 32, 64, 128 μmol/L); 3) MSA group: H9c2 cells were treated with different concentrations of MSA (8, 16, 32, 64, 128 μmol/L); 4) AR group: H9c2 cells were treated with different concentrations of AR (8, 16, 32, 64, 128 μmol/L).

Upon incubation for 24 h, MTT (0.5 mg/mL) was added to each well, followed by a 4-h incubation period inside a light-protected incubator. Subsequently, DMSO was added and the optical density (OD) was measured at 490 nm wavelength. The cell survival was calculated using the relevant Formula 1.

$$\text{Cell survival }\left(\%\right)=\left(\text{experiment hole OD }/\text{ blank hole OD}\right)\times 100\%$$ (1)

#### 2.4.3 Cell viability of triterpenoids on MA-induced H9c2 cardiomyocyte

We conducted the MTT assay to evaluate the ability of triterpenoids from CF to attenuate cardiac toxicity induced by MA. The best-attenuated concentration was identified and used in subsequent experiments. The MTT assay was performed as described in Section 2.4.2. They were distributed into four groups as follows: 1) control group: H9c2 cells were cultured for 24 h without any treatment; 2) MA group: H9c2 cells were treated with 300 μmol/L of MA for 24 h; 3) MA+CRA group: H9c2 cells were treated with 300 μmol/L of MA and different concentrations of CRA (2, 4, 8, 16, 32 μmol/L) for 24 h; 4) MA+MSA group: H9c2 cells were treated with 300 μmol/L of MA and different concentrations of MSA (2, 4, 8, 16, 32, 64 μmol/L) for 24 h; 5) MA+AR group: H9c2 cells were treated with 300 μmol/L of MA and different concentrations of AR (2, 4, 8, 16, 32, 64, 128 μmol/L) for 24 h.

#### 2.4.4 Nucleus morphology of triterpenoids on MA-induced H9c2 cardiomyocyte

Cell apoptosis was analyzed using Hoechst 33258 staining (Lablead, Beijing, China). H9c2 cells were treated with a medium containing MA or different concentrations of triterpenoids for 24 h. The grouping and dosages were as follows: 1) Control group: H9c2 cells were cultured without any treatment; 2) MA group: H9c2 cells were treated with 300 μmol/L of MA; 3) MA+CRA group: H9c2 cells were treated with 300 μmol/L of MA and different concentrations of CRA (28, 32, 36 μmol/L); 4) MA+MSA group: H9c2 cells were treated with 300 μmol/L of MA and different concentrations of MSA (12, 16, 20 μmol/L); 5) MA+AR group: H9c2 cells were treated with 300 μmol/L of MA and different concentrations of AR (60, 64, 68 μmol/L).

Following the treatment, the cells were stained with the Hoechst 33258 for 20 min in the absence of light. After then observed under a fluorescence microscope.

#### 2.4.5 Mitochondrial membrane potential of triterpenoids on MA-induced H9c2 cardiomyocyte

We assessed changes in mitochondrial membrane potential in H9c2 cells by using JC-1 dye staining (Solarbio, Beijing, China) to confirm the mechanism by which triterpenoids from CF can reduce cardiac damage induced by mesaconitine. H9c2 cells were treated with a medium containing MA or triterpenoids for 24 h. The grouping and dosages were as follows: 1) control group: H9c2 cells were cultured without any treatment; 2) MA group: H9c2 cells were treated with 300 μmol/L of MA; 3) MA+CRA group: H9c2 cells were treated with 300 μmol/L of MA and 32 μmol/L of CRA; 4) MA+MSA group: H9c2 cells were treated with 300 μmol/L of MA and 20 μmol/L of MSA; 5) MA+AR group: H9c2 cells were treated with 300 μmol/L of MA and 64 μmol/L of AR.

Subsequently, the H9c2 cells were treated with JC-1 staining solution. And then incubated for 20 min in a 5% CO_2_ incubator at 37°C. Apoptotic cells were directly visualized with inverted fluorescence microscopy.

### 2.5 Verification of triterpenoids reducing H9c2 cardiomyocyte toxicity induced by MA based on TRPV1 channel

#### 2.5.1 TRPV1 expression in the triterpenoids or MA-treated H9c2 cardiomyocyte

The Western blot assay was performed to validate the results of the previous pharmacophore screening and molecular docking, which screened the triterpenoids with activation activity on the TRPV1 channel. This laid the groundwork for the subsequent confirmation of whether the effect of triterpenoids alleviating cardiotoxicity caused by MA was related to TRPV1.

H9c2 cells were treated with a medium containing MA or triterpenoids for 24 h. The grouping and dosages were as follows: 1) control group: H9c2 cells were cultured without any treatment; 2) MA group: H9c2 cells were treated with 300 μmol/L of MA; 3) CRA group: H9c2 cells were treated with 32 μmol/L of CRA; 4) MSA group: H9c2 cells were treated with 20 μmol/L of MSA; 5) AR group: H9c2 cells were treated with 64 μmol/L of AR.

Following treatment, the H9c2 cells were lysed with the total cell protein extraction reagent and then centrifuged at 13,000 g for 5 min at 4°C. The resulting supernatant collected was the total protein solution. The levels of proteins in the supernatant were determined using a BCA kit (Baiqiandu, Wuhan, China). Afterward, the total protein was separated by SDS-polyacrylamide gel electrophoresis (SDS-PAGE, Baiqiandu, Wuhan, China) and transferred onto polyvinylidene difluoride membranes (PVDF, Millipore, United States). The membranes were treated with the various primary antibodies (GAPDH, 1:6,000; TRPV1, 1:1,000) overnight after being blocked with 5% non-fat dry milk at 37°C for 2 h. After washing the membrane with TBST (Baiqiandu, Wuhan, China), the membranes were incubated with secondary antibodies for 30 min at room temperature. Finally, an ECL chemiluminescent (Baiqiandu, Wuhan, China) working buffer was treated with the protein bands. After that, the bands’ quantitative grayscale analysis was done using the IPWIN60 program.

#### 2.5.2 Intracellular calcium ions content of triterpenoids on MA-induced H9c2 cardiomyocyte

The TRPV1 channel is a calcium ion channel, and it plays a crucial role in the flow of calcium ions into cells. The effect of this channel on calcium ions in cardiomyocytes was detected by Fluo-3 AM fluorescent probe method. The H9c2 cells were treated with a medium containing MA or various doses of triterpenoids for 24 h. Groups allocation and dose were the same as in Section 2.4.5. Following the treatment, the Fluo-3 AM (Solarbio, Beijing, China) (5 μmol/L) was used to stain the cells. The cells were then rinsed with HBBS after being incubated for 60 min at 5% CO_2_ at 37°C, and the fluorescent intensity was assessed using a fluorescence microscope.

#### 2.5.3 Cell viability of triterpenoids on MA-induced H9c2 cardiomyocyte after TRPV1 channel blocked by the capsazepine

To further explore whether the attenuated effect is related to the TRPV1 channel, we observed the effect of triterpenoids on the survival rate of H9c2 cells treated with MA before and after the TRPV1 channel blockade by MTT assay. H9c2 cells were treated with a medium containing MA or triterpenoids for 24 h. The grouping and dosages were as follows: 1) control group: H9c2 cells were cultured without any treatment; 2) MA group: H9c2 cells were treated with 300 μmol/L of MA; 3) triterpenoid + MA group: H9c2 cells were treated with 300 μmol/L of MA and one of the triterpenoids (32 μmol/L of CRA; 20 μmol/L of MSA; 64 μmol/L of AR); 4) Capsazepine + triterpenoid + MA group: H9c2 cells were pretreated with capsazepine (10 μmol/L) for 30 min before 300 μmol/L of MA and one of the triterpenoids (32 μmol/L of CRA; 20 μmol/L of MSA; 64 μmol/L of AR).

### 2.6 Statistical analysis

All of the above cell experiments were independently repeated three times. The GraphPad Prism (version 8.0.1) was utilized to create all statistical calculations and graphs. Two-tailed Student’s t-test was implemented to compare the two groups. ANOVA was used to compare three or more groups against one another. The mean and standard deviation (SD) were employed to express the results. When *p* < 0.05, the differences were deemed statistically significant.

## 3 Results

### 3.1 Pharmacophore model screening

#### 3.1.1 Pharmacophore model establishment and validation

**FIGURE 2:**
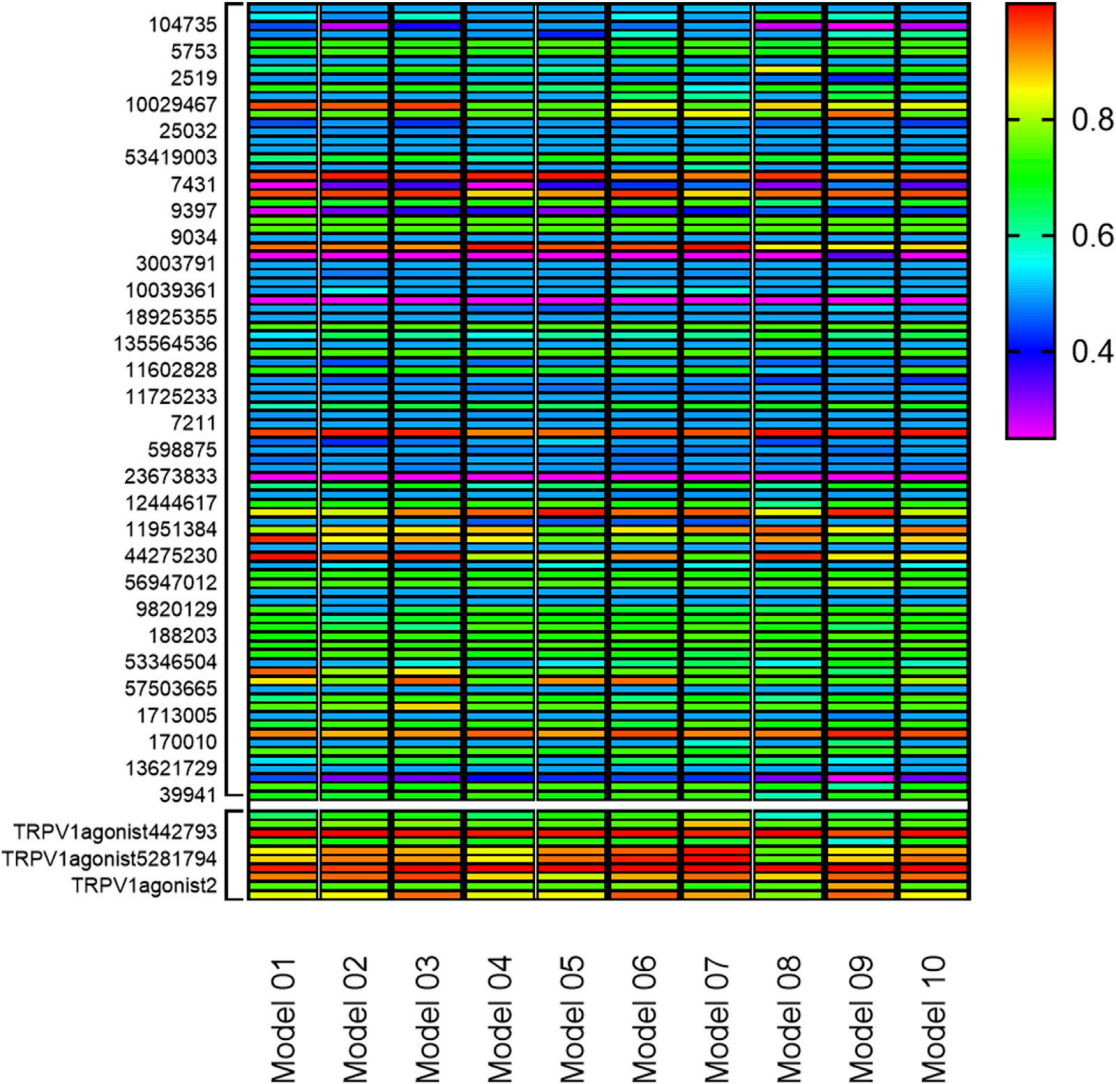
Validation of 10 pharmacophore models. (The bottom 10 color blocks in each column correspond to the TRPV1 agonists, and the rest 90 correspond to the components that have not been verified for activity. The ligands that depict higher responses to the pharmacophore models have a warm tone while those with lower responses have a cold tone.)

Based on the commonly shared features amongst seven agonists of TRPV1 selected from the training set, ten pharmacophoric models were constructed and their pharmacophoric characteristics along with Rank values were reported in Table 1. All the pharmacophores contained four features, namely, A: ACCEPTOR (Hydrogen bond acceptor) and H: HYDROPHOBIC had two each. The Rank values of these ten pharmacophoric models were greater than 60, signifying the prominence of the molecular common features while constructing the pharmacophores. Additionally, the differences among the top scores were extremely small. Hence, it was imperative to validate and screen the generated 10 pharmacophore models using a test set (Figure 2). The ligands that depict higher responses to the pharmacophore models have a warm tone and those with lower responses have a cold tone. As seen, pharmacophore model 02 portrayed high responses against the active ingredients present in the test set and relatively lower responses to the inactive ingredients, thereby meeting the study requirements. Hence, pharmacophore model 02 was selected for virtual screening.

**TABLE 1:** Results of pharmacophore models construction.

No.	Features	Max fit	Rank value
01	HHAA	4	68.372
02	HHAA	4	67.154
03	HHAA	4	66.907
04	HHAA	4	66.849
05	HHAA	4	66.170
06	HHAA	4	66.168
07	HHAA	4	65.651
08	HHAA	4	65.077
09	HHAA	4	64.964
10	HHAA	4	64.007

The highest number of features is 4. H (hydrophobic features), A (Acceptor).

Max Fit represents the matching situation of the pharmacophore features.

Rank Value indicates the score of the pharmacophore, the higher the score, the better.

#### 3.1.2 Virtual screening based on pharmacophore model 02

**FIGURE 3:**
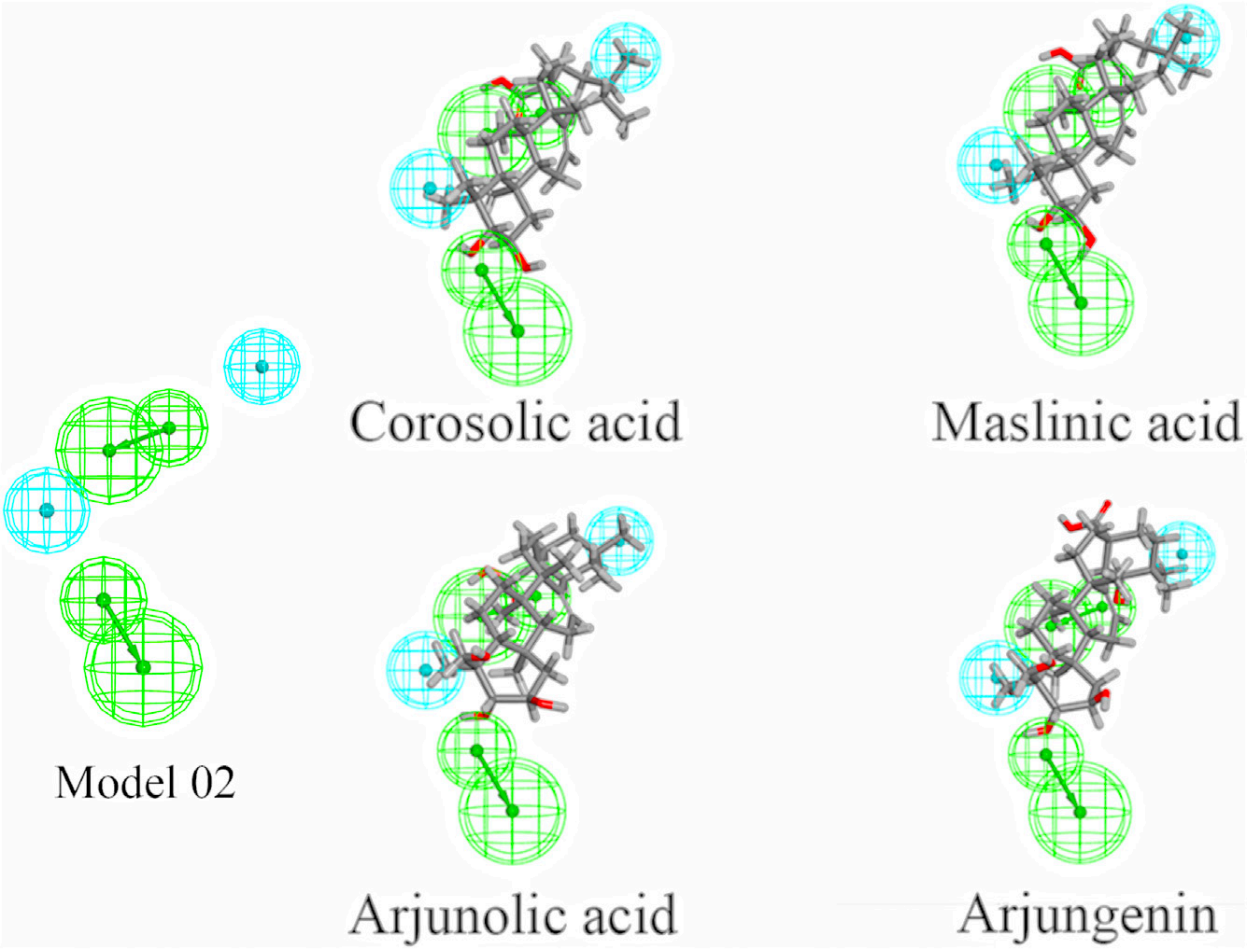
Pharmacophore model 02 and mapping with the selected triterpenoids. (The green spheres represent hydrophobic features, while blue spheres represent hydrogen bond acceptor features.)

The 60 components in CF were virtually screened using the pharmacophore model 02 selected in Section 3.1.1, and eight components were finally selected (Table 2), comprising seven triterpenoids and one alkaloid. Furthermore, the matching diagram of the pharmacophore and some potential active components were shown in Figure 3. An observation from the figure depicted that the triterpenoids exhibited similar structural features as that of the pharmacophore model.

**TABLE 2:** Compounds of the CF selected by pharmacophore model 02 with potential activation of TRPV1.

NO.	Compound	Fit value
1	Catharanthamine	2.76795
2	Corosolic acid	2.28106
3	Chebupentol	1.86848
4	Arjungenin	1.79283
5	Arjunolic acid	1.78225
6	Maslinic acid	1.50544
7	Terminolic acid	1.46320
8	Chebuloside-Ⅱ	1.29666

Fit Value indicates the matching status of the components to the pharmacophore, the larger the value, the better the matching degree.

### 3.2 Molecular docking analysis

#### 3.2.1 Molecular docking validation

When assessing the stability of the molecular docking model, re-docking the co-crystallized ligand into the empty binding pocket of the protein was employed. The calculated pose was then compared to the bioactive conformation from the crystal structure and the root mean square distance (RMSD) was calculated. Typically, RMSD values below 2 Å are considered acceptable (Temml and Kutil, 2021). Upon re-docking, the structural and spatial positions were fairly consistent with the native ligand. The RMSD (5IRX) = 1.2826 Å < 2 Å, RSMD (5IS0) = 1.7092 Å < 2 Å. Lower Score indicates higher affinity towards the TRPV1 receptor and greater propensity for interaction. The outcome (Table 3) exhibits that most of the other small molecule ligands displays high affinity and are in close proximity to the aforementioned ligands. This affirmed the high reliability of the molecular docking method utilized in this study. Meanwhile, The docking data also provides reference values for the subsequent molecular docking simultaneously.

**TABLE 3:** Docking scores of TRPV1 agonists and TRPV1 antagonists with 5IRX and 5IS0 respectively (Kcal/mol).

NO.	TRPV1 agonist	Scores (5IRX)	NO.	TRPV1 antagonist	Scores (5IS0)
1	Resiniferatoxin (RTX)	−9.8	1	MK-2295	−9.9
2	Compound 3	−7.9	2	AMG-517	−9.6
3	DA-5018	−6.1	3	SB-705498	−9.5
4	Compound 2	−8.4	4	K-685	−9.2
5	piperine	−8.3	5	ABT-102	−9.2
6	Compound 1	−8.1	6	Capsazepine	−9.1
7	capsaicin	−7.0	7	BCTC	−8.8
8	6-Gingerol	−7.0	8	PAC-14028	−8.4
9	zingerone	−6.5	9	SB-366791	−8.2
10	6-shogaol	−7.0	10	SC-0030	−7.4

The smaller binding affinity indicates a higher affinity for the TRPV1 receptor.

#### 3.2.2 Virtual screening based on molecular docking

**FIGURE 4:**
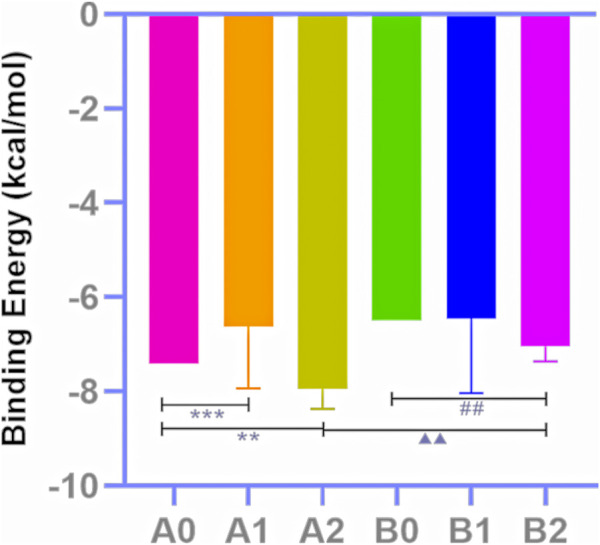
Docking score analysis. Molecular docking results of MA (A0), CF all components (A1), triterpenoids selected by the pharmacophore model 02 (A2) with 5IRX; molecular docking results of MA (B0), CF all components (B1), triterpenoids selected by the pharmacophore model 02 (B2) with 5IS0 (Compare A2 to A0, **p < 0.01; compare A2 to A1, ^##^ *p* < 0.01; compare B2 to A2, ^△△△^ *p* < 0.001).

The docking outcomes of CF components with TRPV1 receptors were presented in Supplementary Table S4. All of them and MA docking results were scrutinized and subsequently compared (Table 4; Figure 4). The results indicates that pharmacophore-selected triterpenoids exhibits higher affinity towards TRPV1 receptor 5IRX than MA (**p < 0.01) as well as other CF components (^##^ *p* < 0.01). Furthermore, these constituents demonstrates a higher affinity for the 5IRX receptor than the 5IS0 receptor (^∆∆∆^ *p* < 0.001).

**TABLE 4:** Docking scores of mesaconitine and the selected triterpenoids by the pharmacophore with 5IRX and 5IS0 (Kcal/mol).

NO.	Compound	Scores (5IRX)	Scores (5IS0)
1	Corosolic acid	−8.5	−7.4
2	Chebuloside-Ⅱ	−8.4	−7.1
3	Terminolic acid	−8.1	−7.1
4	Maslinic acid	−8.0	−7.3
5	Arjungenin	−7.7	−7.0
6	chebupentol	−7.5	−6.4
7	Arjunolic acid	−7.4	−7.0
8	Mesaconitine	−7.4	−6.5

### 3.3 Activity prediction of triterpenoids and MA based on 3D-QSAR model

#### 3.3.1 3D-QSAR model construction and validation

**FIGURE 5:**
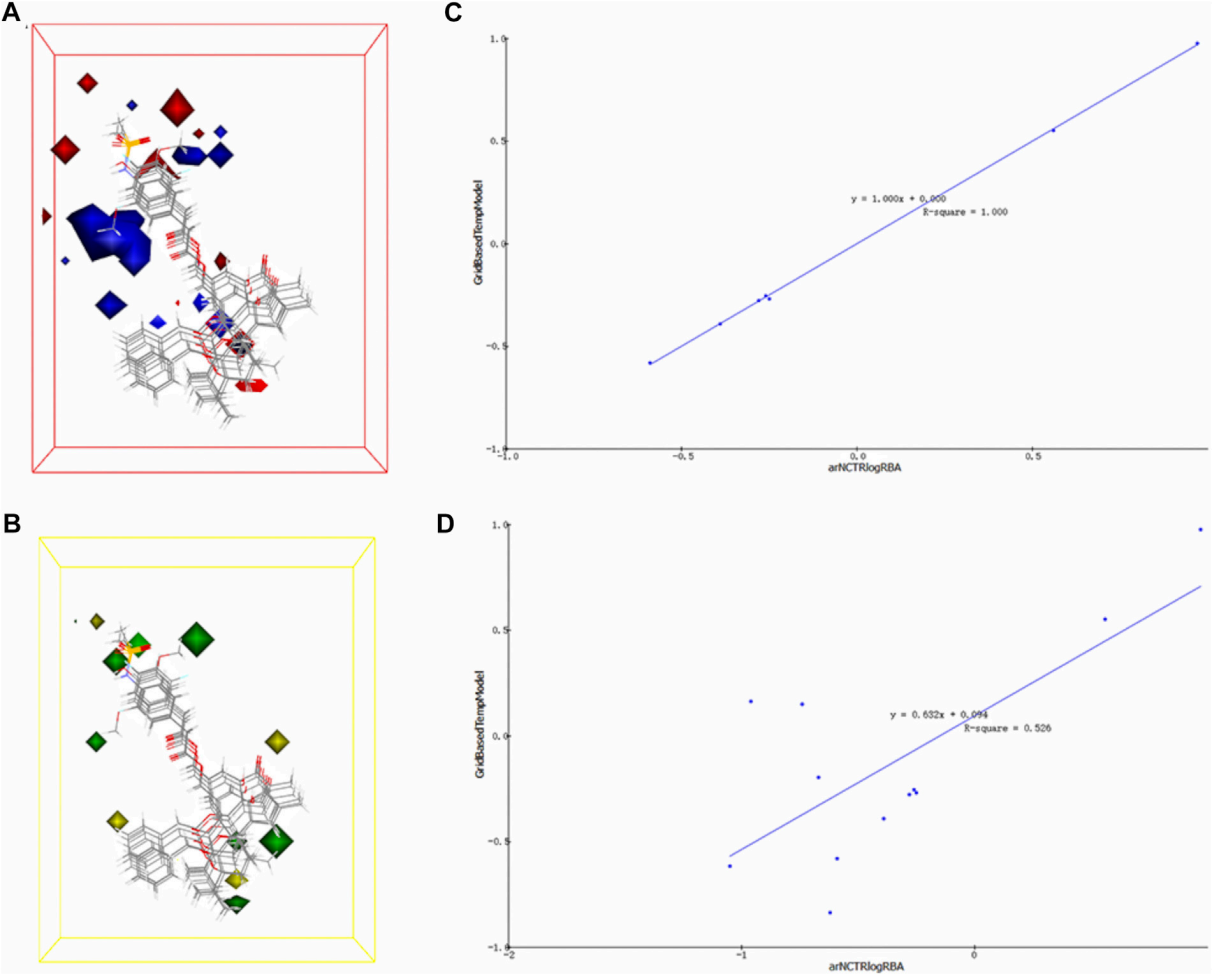
3D-QSAR model electrostatic and stereo field coefficient contour maps. **(A)** the high map of electrostatic field coefficient. **(B)** the high map of stereo field coefficient. **(C)** The fitting diagram of the prediction value of the 3D-QSAR model and the experimental value based on the training set. **(D)** The fitting diagram of the prediction value of the 3D-QSAR model and the experimental value based on the test set.

The 3D-QSAR model is a pharmacophore model with activity prediction ability based on a series of compounds with clear activity values for specific biological targets. The model constructed in this section was based on the electrostatic field and steric field characteristics of TRPV1 agonists, which can be used to predict the activity of unknown compounds, thus providing a basis for subsequent experimental verification and mechanism exploration (Figures 5A, B). In the electrostatic map, the red area indicates that high negative charges enhance the compound’s activity, while the blue area suggests high positive charges improve the compound activity. In the stereo field map, the yellow region denotes that expanding this area’s volume does not enhance the compound activity, while the green area indicates that increasing the region’s volume improves the compound activity.

The training set’s predicted activity values were compared to experimental activity values (Figure 5C), with the *R* ^*2*^ value reflecting the accuracy of the curve. The curve’s *R* ^*2*^ value of 1, indicating accurate prediction and closely matching experimental values.

To verify the accuracy of the 3D-QSAR model, the fitted curve based on the prediction and experimental values of the test set was created (Figure 5D). The *R* ^*2*^ value of 0.526 indicates that the model has some confidence, with the predicted values reflecting the experimental values to a reasonable extent. The model has the potential to predict the activity order of triterpenoids and MA’s potential as a TRPV1 agonist.

#### 3.3.2 Results of activity prediction based on the 3D-QSAR model

The developed 3D-QSAR model was utilized for predicting the activity of triterpenoids and MA, as shown in Table 5. All of the eight components in the table exhibited activity, among which MA had the strongest predicted activity with a predicted pEC_50_ of −0.446712. The triterpenoids exhibited relatively weak activity, with CRA, MSA, and AR exhibiting the weakest activity among them.

**TABLE 5:** The prediction results of mesaconine and triterpenoids as TRPV1 agonist activity.

NO.	Compound	pEC_50_
1	chebupentol	−0.356389
2	Mesaconitine	−0.446712
3	Terminolic acid	−0.503938
4	Arjunolic acid	−0.519541
5	Chebuloside-Ⅱ	−0.528765
6	Arjungenin	−0.580258
7	Corosolic acid	−0.587511
8	Maslinic acid	−0.618522

### 3.4 Verification of triterpenoids alleviating the H9c2 cardiomyocyte toxicity caused by MA

#### 3.4.1 Effect of triterpenoids on H9c2 cell viability

**FIGURE 6:**
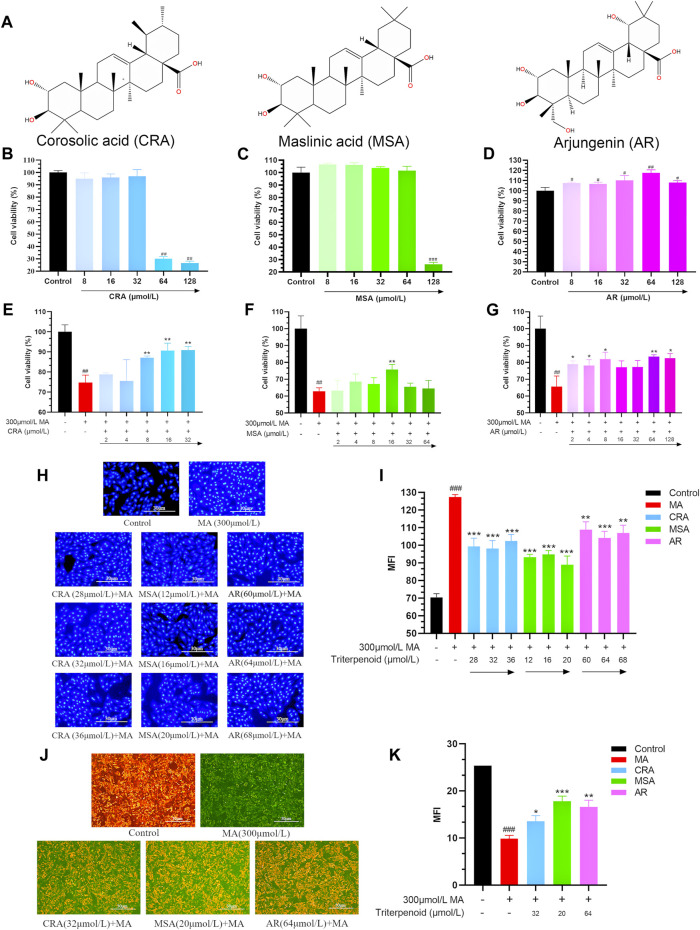
Triterpenoids attenuated cardiomyocyte toxicity caused by MA. **(A)** The structure of selected triterpenoids. Effect of CRA **(B)**, MSA **(C)**, and AR **(D)** on cell viability by MTT assay. The attenuating effect of CRA **(E)**, MSA **(F)**, and AR **(G)** on MA caused myocardial cytotoxicity by MTT assay. Effect of triterpenoids or MA on cell nucleus apoptosis in H9c2 cells by Hoechst 33258 staining, **(H)** fluorography (×100); **(I)** quantification is expressed as the Mean fluorescence indensity (MFI). Effect of triterpenoids or MA on mitochondrial membrane potential in H9c2 cells by JC-1 dye staining, **(J)** fluorography (×100); **(K)** quantification is expressed as the MFI. Data were presented as mean ± SD (*n* = 3. Compared with normal control group, ^#^ *p <* 0.05, ^##^ *p* < 0.01; compared with MA group, *p < 0.05, **p < 0.01, ***p < 0.01).

The structures of the three triterpenoids were shown in Figure 6A. As shown in Figures 6B–D, the cell survival rate decreased when the CRA was in the range of 64 μmol/L to 128 μmol/L and MSA at 128 μmol/L (^##^ *p* < 0.01, ^###^ *p* < 0.001). In contrast, AR not only exhibited safety and non-toxicity towards H9c2 cells in the concentration range of all 128 μmol/L, but also promoted cell growth (^#^ *p* < 0.05), and the proliferation reached its maximum effect at 64 μmol/L of AR (^##^ *p* < 0.01). For subsequent experimental verification, concentration ranges of 0 ∼ 32 μmol/L, 0 ∼64 μmol/L, and 0 ∼ 128 μmol/L were selected for CRA, MSA, and AR, respectively.

#### 3.4.2 Effect of triterpenoids on MA-induced cell viability in H9c2 cardiomyocyte

To confirm the triterpenoids can lessen the H9c2 cardiomyocyte toxicity brought on by MA, the survival rate of H9c2 cells was examined using the MTT assay (Figures 6E–G). MA (300 μmol/L) significantly decreased the survival rate of H9c2 cells as compared to the control group (^##^ *p* < 0.01). All three triterpenoids have the potential to reduce the toxicity of MA on cardiomyocytes and increase cell survival (32 μmol/L of CRA, 16 μmol/L of MSA, 64 μmol/L of AR, **p < 0.01). And the optimized attenuated concentration were selected for subsequent experiments.

#### 3.4.3 Effect of triterpenoids on MA-induced nuclear apoptosis in H9c2 cardiomyocyte

The cells nuclear change was obtained by Hoechst 33258 staining (Figures 6H, I). Hoechst 33258 specifically stains the nucleus, and when the cell becomes apoptotic, its nuclei is colored blue and fragmented. The control group’s nucleus displayed light blue fluorescence following staining and the nucleus shape were unaltered in the Hoechst 33258 staining results. Compared to the control group, the nucleus’ apoptotic features became visible in the MA group. It was obviously that the nucleus’ chromatin was compressed, and some fragments could be observed. The average fluorescence intensity was much higher than that of the control group on the entire, and it displayed dense and strong bright blue fluorescence (^###^ *p* < 0.001). In comparison to the MA group, the addition of a small amount of CRA, MSA, and AR greatly enhanced cell apoptosis, dramatically enhanced nuclear chromatin condensation, and lowered fluorescence intensity (***p < 0.001, **p < 0.01).

#### 3.4.4 Effect of triterpenoids on MA-induced mitochondrial membrane potential in H9c2 cardiomyocyte

The cells mitochondrial membrane potential change was measured by JC-1 staining (Figures 6J, K). During the JC-1 staining experiment, healthy cells produce red fluorescence due to their higher mitochondrial membrane potential. Conversely, apoptotic cells produce green fluorescence due to their lower membrane potential. A decrease in mitochondrial membrane potential is an early sign of apoptosis, and this decrease can be detected easily by the transition from red fluorescence to green fluorescence when cellular membrane potential drops. In the control group, orange-red fluorescence was predominantly observed, with only a small amount of green fluorescence indicating higher mitochondrial membrane potential. The MA group showed decreased mitochondrial membrane potential, as indicated by greater green fluorescence and weaker orange-red fluorescence (^###^ *p* < 0.001). In comparison to the MA group, the inclusion of triterpenoids significantly increased mitochondrial membrane potential (***p < 0.001, *p < 0.01, *p < 0.05).

Through this part of the experiment, we selected the concentrations of CRA, MSA, and AR as 32 μmol/L, 20 μmol/L, and 64 μmol/L for the subsequent exploration of the mechanism of triterpenoids reducing H9c2 cardiomyocyte toxicity induced by MA.

### 3.5 Verification of triterpenoids reducing MA-induced H9c2 cardiomyocyte toxicity via TRPV1 channel

#### 3.5.1 Effect of triterpenoids or MA on TRPV1 expression in H9c2 cardiomyocyte

**FIGURE 7:**
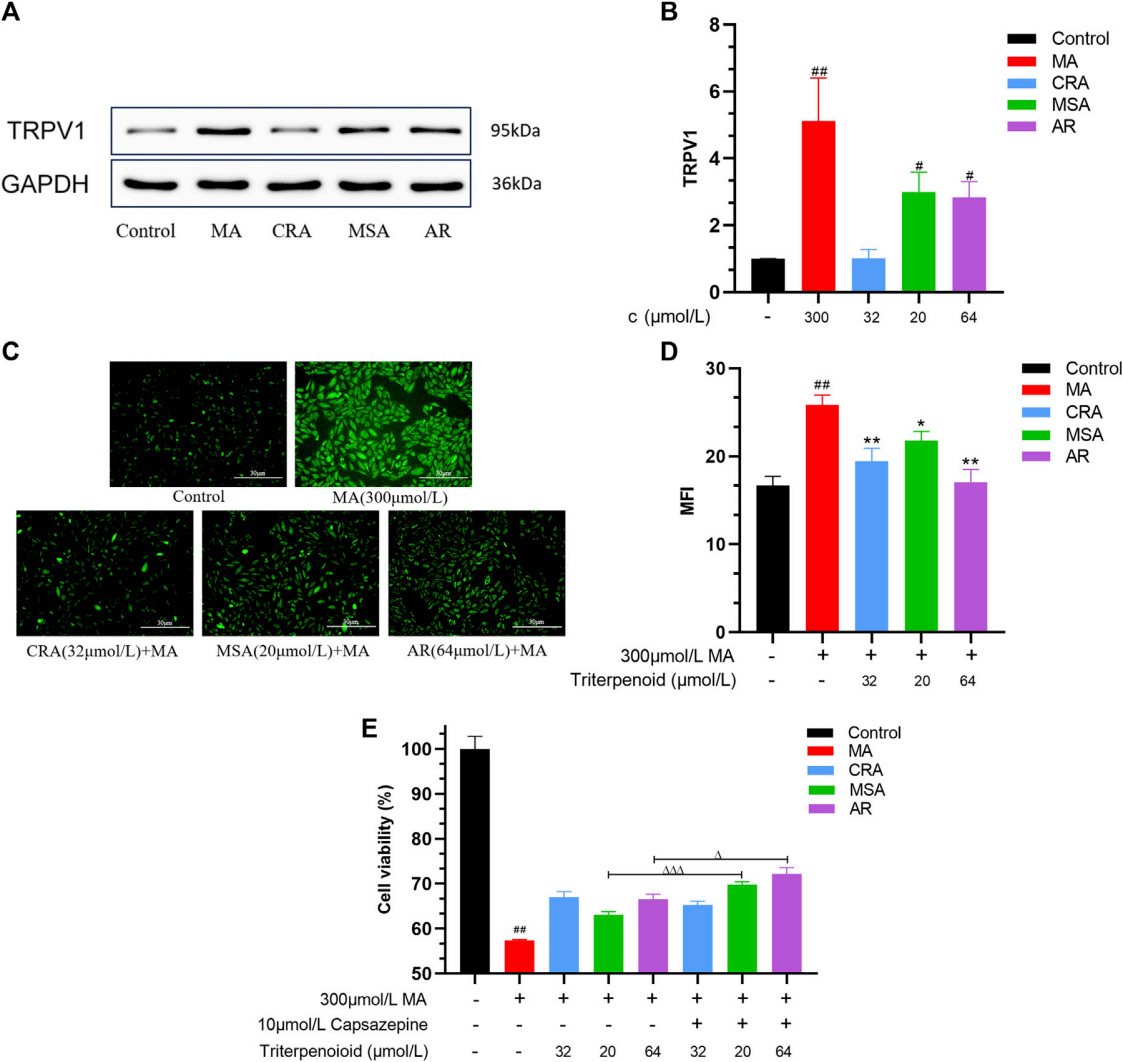
Triterpenoids attenuated cardiomyocyte toxicity caused by MA through TRPV1 channel. Effect of triterpenoids or MA on expression of TRPV1 in H9c2 cells detected by Western blot, **(A)** Western blot result, **(B)** the semiquantitative result of **(A)**. Effect of triterpenoids or MA on Ca^2+^ concentration in H9c2 cells by Fluo-3 AM, **(C)** fluorography (×100); **(D)** quantification is expressed as the MFI. **(E)** Effect of triterpenoids or MA on cell survival before and after TRPV1 channel blockade by MTT assay and capsazepine. Data were presented as mean ± SD (*n* = 3. Compared with normal control group, ^#^ *p* < 0.05, ^##^ *p* < 0.01; compared with MA group, *p < 0.05, **p < 0.01, ***p < 0.001; compared with the group of the TRPV1 channels that were not blocked, ^△^ *p* < 0.05, ^△△△^ *p* < 0.001).

As depicted in Figures 7A, B, MA, MSA, and AR significantly increased the expression of the TRPV1 protein in H9c2 cells (**p < 0.01, ***p < 0.001), although MSA and AR had a weaker effect when used in attenuated toxicity concentrations. CRA specifically failed to induce TRPV1 protein expression.

#### 3.5.2 Effect of triterpenoids on MA-induced intracellular calcium ions content in H9c2 cardiomyocyte

The changes in calcium ion concentrations in H9c2 cells were estimated. As illustrated in Figures 7C, D, the intracellular Ca^2+^ level was lower in the control group, and only a trace of green fluorescence was observed. However, MA may greatly boost the green fluorescence intensity of H9c2 cells as well as the intracellular Ca^2+^ content (^*###*^ *p* < 0.001). The addition of CRA, MSA, and AR significantly reduced both the green signal and intracellular Ca^2+^ levels compared to the MA group (*p < 0.05, **p < 0.01).

#### 3.5.3 Effect of triterpenoids on MA-induced H9c2 cardiomyocyte toxicity after TRPV1 channel blocked by the capsazepine

MA dramatically decreased the H9c2 cells’ survival rate as compared to the control group (^#^ *p* < 0.01) (Figure 7E). Compared to the group without capsazepine, the AR and MSA groups that had previously received capsazepine were able to further improve the survival rate of the H9c2 cells (^∆^ *p* < 0.05, ^∆∆∆^ *p* < 0.001), but the CRA group was not significantly different regardless of whether they had received capsazepine in advance treatment.

## 4 Discussion

Aconitum herbs have a long history of medication, such as *Aconitx kusnezoffll* radx., and *Aconitum pendulum* Busch. They have good anti-inflammatory and analgesic properties, but their toxicity is also extremely strong (Singhuber et al., 2009). It is necessary to study the measures and mechanism of toxicity reduction of aconitums, which is of great significance for safe drug use in clinical practice. In Mongolian and Tibetan medicine theory, CF is often used to process or combine aconitums to reduce their toxicity (Liu et al., 2017; Liet al., 2022b). Studies have shown that CF has the effect of reducing the toxicity of aconitums (Liu et al., 2015a; Zhang et al., 2017; Yuan et al., 2022), and the mechanism of the attenuation toxicity was not the same as the conventional heating and boiling to convert the toxic DDAs into less toxic and more powerful monoester-diterpenoid alkaloids (MDAs) or non-esterified diterpene alkaloids (NDAs) (Liu et al., 2013), involving multiple pathways *in vitro* and *in vivo*, such as material basis, internal absorption and metabolism, myocardial electrophysiology, etc. (Zhang, 2019; Tan et al., 2023). On the basis of the previous studies, we aimed to clarify the mechanism of reducing cardiotoxicity from the aspect of TRPV1 channel that DDAs induced cardiotoxicity in this study.

The pharmacophore model, molecular docking and 3D-QSAR model were used for virtual screening in this study. To screen for components in CF that may activate the TRPV1 channel, a pharmacophore model based on common features of TRPV1 agonists was constructed. However, the pharmacophore model can only identify components with activation potential; it cannot determine their relative activity or ability to bind to the TRPV1 protein. Furthermore, molecular docking was used to quantify each component’s binding capacity to the TRPV1 protein receptor, which served as a confirmation and secondary screening of the pharmacophore results. However, further clarification of their activities is required. Consequently, a 3D-QSAR model based on TRPV1 agonists was developed to predict the activity of the screened chemical components. The pharmacophore model revealed seven triterpenoids in CF with TRPV1 agonistic effects. Subsequently, the molecular docking and 3D-QSAR model results indicated that the triterpenoids of CF, which were screened by pharmacophore, had a higher affinity to bind to the TRPV1 agonist receptor than MA. However, their relative activity was lower than that of MA, with CRA, MSA, and AR exhibiting the lowest predicted activity. Thus, we initially integrated the results of the virtual screening section and speculated that the triterpenoids in CF function as TRPV1 agonists. These compounds have a higher affinity for TRPV1 receptors and can competitively antagonize the binding of MA to TRPV1 receptors, whereas having lower activity. This prevents the over-activation of TRPV1 channels and exerts an attenuated effect.

The rat H9c2 cardiomyocytes were then used to validate the results of the virtual screen. The administration concentration of MA was determined according to the previous pre-experiments. The concentrations of triterpenoids were selected step by step during the experiment. First, the triterpenoids were screened for safe and non-toxic ranges. Then the best attenuation was screened in the attenuated verification experiment section by degrees. Obviously, MA damaged the viability of H9c2 cardiomyocytes, seriously reduced the survival rate. It also caused nuclear damage and pyknosis, and decreased mitochondrial membrane potential. These are landmark events in the early stage of apoptosis and also prove the cardiotoxicity of MA. Moreover, MA promoted the expression of TRPV1 protein in cardiomyocytes, which in turn significantly increased the content of calcium ions in cardiomyocytes. This was consistent with the previous study that the MA had myocardial cytotoxicity, and the generation of toxicity was related to the excessive activation of TRPV1 channels. It was also established that the triterpenoids could reduce the myocardial cytotoxicity of MA to some extent, increase the mitochondrial membrane potential and relieve the damage to the nucleus caused by MA. In addition, MSA and AR can similarly stimulated TRPV1 protein expression in H9c2 cells, although their capacities were inferior to that of MA. In addition, the H9c2 cells survival rate increased when TRPV1 channels were blocked by capsazepine, indicating that the attenuated effects of MSA and AR were related to TRPV1 channels and acted as agonists of TRPV1 channels. Not only MSA and AR but also CRA, could attenuate this tendency for large calcium influx caused by MA and maintain homeostasis of intracellular calcium. These tests provided additional evidence that the TRPV1 channel played a role in reducing cardiotoxicity caused by MA, as the TRPV1 channel is a crucial channel for calcium ions to flow into cells.

So far, efforts have been devoted to the study of the mechanism of CF reducing the toxicity of aconitums, with the expectation of providing theoretical support for the safe application of aconitums. The mechanisms can be summarized in four categories: 1) The acidic components in the CF can inhibit the dissolution of DDAs and detoxify the aconitums (Liu et al., 2013; Yang et al., 2013; Xin et al., 2014). 2) Tannins in CF complexed with DDAs in aconitums to form insoluble substances, which slows down the dissolution rate of alkaloids in gastric and intestinal fluids and plays a certain role in reducing toxicity (Liu et al., 2015b). 3) CF can play a role in reducing toxicity by affecting the absorption, distribution and metabolic process of aconitums in the body. For absorption: the pharmacokinetics of alkaloids can be changed after the processing of aconitums or the compatibility with CF, and the absorption level and absorption rate of alkaloids are reduced, thus reducing the toxicity (Zhi et al., 2020b; Liu et al., 2023). For distribution: CF can reduce the distribution of DDAs in various organs and tissues, and the tannins can bind to DDAs and slowly distribute and eliminate them in the body to play a role in detoxification (Wang et al., 2002). Tannic acid in can reduce the plasma protein binding rate and bioavailability of DDAs (Zhang, 2019). For metabolism: CF can change the absorption and metabolism sites of toxic components, reduce its absorption in the intestinal wall, and increase intestinal bacteria and liver metabolism. This can prevent the absorption of toxic components from being too fast, and promote the metabolism, reduce the concentration of drugs in the blood and prevent poisoning (Celimuge et al., 2022). 4) The components in CF have myocardial protective effect, which can effectively inhibit the cardiotoxicity of aconitums. The use of CF can reduce the myocardial tissue damage caused by the single administration of aconitums in rats (Zhang et al., 2017). Scholars have proved that CF had a good intervention effect on arrhythmia caused by aconitine by Langendorff isolated heart perfusion technique (Liang et al., 2017). CF can resolve the toxicity of Aconitums and has a protective effect on the heart (Pan et al., 2011).

CF alleviates the cardiotoxicity of aconitums mainly due to the active ingredients in CF, such as tannins and triterpenoids, protect the heart from the serious toxicity of aconitums through various ways *in vivo*. Researchers have found that CF could regulate the contraction and relaxation of myocardial cells by multi-component, multi-target, multi-channel, further protect the normal function of the heart, and reduce the cardiac toxicity caused by aconitiums by using the method of network pharmacology (Li et al., 2018). Metabolomics technology was used to investigate the effect of CF on endogenous metabolites in rats. The results showed that glutamine and lysophosphatidylcholine may be the key to the detoxification and regulation efficacy of CF, and also have a certain role in improving cardiac function and acute myocardial infarction (Bilige et al., 2022). The combination of ellagic acid, liquiritin and aconitine can significantly increase the expression of cardiac metabolic enzyme CYP2J3, promote the metabolism of arachidonic acid to produce epoxyeicosatrienoic acids, and then reduce the cardiotoxicity (Li et al., 2023). The effective components of CF can inhibit the release of lactate dehydrogenase and creatine kinase isoenzyme and promote the release of superoxide dismutase to achieve the protective effect on aconitine-injured cardiomyocytes (Duolan, 2022). The compatibility of CF with aconitums can regulate the levels of FKBP1B and RyR2 genes in sarcoplasmic reticulum by affecting TRP gene, especially TRPM8, so as to protect myocardium from damage and play a role in reducing toxicity (Meng et al., 2023). Our research group found that Caowu could induce cardiotoxicity by activating the TRPV1 channel, while CF also played an attenuated role through the TRPV1 channel. The attenuated effect may be related to the synergistic effect of the acidic components in CF and the alkaloids in Caowu on the TRPV1 channel (Han et al., 2022c). Subsequently, we used the uniform design method to identify the primary components responsible in CF for the detoxification effect, and then gallic acid and ellagic acid were screened and the two a synergistic effect (Han et al., 2022a). We also demonstrated that chebulagic acid in CF has a protective effect on myocardial mitochondria, and can inhibit aconitine-induced Ca^2+^ overload in myocardial cells, thereby reducing the occurrence of arrhythmia (Han et al., 2020). In addition, we also found that the TRPV1 channel have an important role in exerting their attenuated effects, the gallic acid in CF may achieve the effect of detoxification by rapid desensitization of TRPV1 channel on the cells (Han et al., 2022b; Mi et al., 2023).

In summary, the research on the active ingredients reducing cardiotoxicity caused by aconitums mainly focused on tannins and phenolic acids. These components play a mitigating cardiotoxicity role by affecting the expression of relevant genes, enzyme activity, and ligand-receptor interactions to maintain a steady state within the cardiomyocyte and protect the organelles to maintain normal cellular function. In addition to tannins, the triterpenoids in CF are also noteworthy, and most of them have cardiac and myocardial protective effects. MSA has a protective impact against cardiomyocyte damage by exerting both an anti-oxidative action and an ability to inhibit calcium overload in cardiomyocytes (Liu et al., 2008). The cardioprotective effect of arjunolic acid may lie in its protective potential against damage caused by myocardial necrosis (Sumitra et al., 2001). CRA not only upregulates antioxidant levels in cardiomyocytes and hence inhibits apoptosis and cardiomyocyte stress, but also effectively inhibits the development of fibrosis after myocardial infarction, thereby improving cardiac function (Wang, 2019; An et al., 2020). They are in line with how DDAs in Aconitum herbs exert cardiotoxicity. It was also discovered that the triterpenoids in CF showed a certain potential for TRPV1 activation, which may play an attenuated role (Mi et al., 2023).

Combined with the virtual screening and the attenuated validation experiment, the results preliminarily demonstrated that the triterpenoids in CF played a certain role in CF reducing aconitums cardiotoxicity. MSA and AR most likely acted as TPPV1 agonists with less potency that acted on the TRPV1 channel in cardiomyocytes like MA. Because of their high binding capacity, they can bind better to TRPV1 channels but exert weaker agonism, thus preventing MA from excessively stimulating the TRPV1 channel, inhibiting the large influx of calcium ions, keeping cells viable and achieving the purpose of attenuating cardiotoxicity. Besides, it was discovered that CRA failed to increase the expression of TRPV1 protein on its own, and its attenuating effect did not change before and after capsazepine blocked the TRPV1 channel. Nevertheless, CRA was still able to alleviate the calcium ion influx caused by MA. In this context, it is hypothesized that CRA did not achieve an attenuated effect through the TRPV1 channel, but it was likely through other channels related to calcium ions, such as the MAPK pathway (Schaub et al., 2006; Ai et al., 2016), and we will also explore this in depth next.

The toxicity and efficacy of aconitums are closely related to the TRPV1 channel, so reducing their cardiotoxicity through this channel while maintaining good efficacy is very meaningful. In this study, based on the theory of compatibility or processing in Traditional Chinese Medicine Science, the mechanism of reducing the toxicity of MA by triterpenoids of CF was elucidated from the perspective of antagonistic or synergistic effects of active components of drugs. At present, we have only explored the mechanism through computer simulation and at the cellular level. Next, we will further investigate the mechanism of attenuation at the animal level. Of course, other pathways also play a role in the reduction process, and the link and leading role between them are unclear. The DDAs toxicity is produced by the TRPV1 channel, and its analgesic anti-inflammatory effect is also related to TRPV1 channel. We only have evidence that triterpenoids can reduce MA toxicity at the cardiomyocyte level, but we are not clear about their treatment effect. So how can reduce the toxicity and at the same time show good treatment effects needs to further explored.

## 5 Conclusion

The triterpenoids in CF can reduce the H9c2 cardiomyocyte toxicity caused by MA. Through excessive TRPV1 channel activation, MA causes significant cardiomyocyte death, while MSA and AR also exert a detoxifying effect on cells via TRPV1 channels. Due to their higher affinity with TRPV1 protein receptors, they can competitively antagonize the binding of MA to TRPV1 channels, but at the same time, they have lower TRPV1 channel activation activity than MA, thus preventing MA from overactivating TRPV1 channels, thereby protecting cardiomyocytes and reducing toxicity. CRA may play an attenuated role through other pathways that affect intracellular calcium homeostasis. This paper provides ideas and credence to investigate the mechanism of CF alleviating the cardiotoxicity caused by aconitums *in vitro* experiments.

## Data Availability

The original contributions presented in the study are included in the article/Supplementary Material, further inquiries can be directed to the corresponding author.

## Abbreviation

AR, Arjungenin; CF, Chebulae Fructus; CRA, Corosolic acid; DDAs, Diesterditerpenoid alkaloids; DMEM, Dulbecco’s modified eagle medium; DMSO, Dimethyl sulfoxide; EC50, Concentration for 50% of maximal effect; FBS, Fetal bovine serum; LD50, Median lethal dose; MA, Mesaconitine; MDAs, Monoester-diterpenoid alkaloids; MFI, Mean fluorescence indensity; MSA, Maslinic acid; MTT, 3-(4,5-Dimethylthiazol-2-yl)-2,5-diphenyltetrazolium bromide; NDAs, Non-esterified diterpene alkaloids; OD, Optical density; RMSD, Root-mean-square deviation; RTX, Resiniferatoxin; SD, Standard deviation; TRPV1, Transient receptor potential vanilloid type 1 channel; WB, Western blot.
