# Supplementary material for: Integrated virtual screening and in vitro studies for exploring the mechanism of triterpenoids in Chebulae Fructus alleviating mesaconitine-induced cardiotoxicity via TRPV1 channel
Source: Front Pharmacol. 2024 Mar 4;15:1367682. doi: 10.3389/fphar.2024.1367682 (PMC10945000; doi:10.3389/fphar.2024.1367682)
Supplement: Supplementary file 1 [file Table1.DOCX]

Supplementary Material

Integrated virtual screening and in vitro studies for exploring the mechanism of triterpenoids in Chebula Fructus alleviating mesaconitine-induced cardiotoxicity via TRPV1 channel

Liangliang Song^1^, Shuo Mi^1^, Ying Zhao^1^, Ziqin Liu^1^, Jing Wang^1^, Hongyue Wang^1^, Wenhui Li^1^, Jiasheng Wang^1^, Wenting Zu^1^, Hong Du^1*^

**^1^**School of Chinese Materia Medica, Beijing University of Chinese Medicine, Beijing 102488, China

*** Correspondence:** Hong Du: duhong@vip.163.com

**Table S1.** Chemical compositions of CF.

| NO. | Compound | Formula | CAS | NO. | Compound | Formula | CAS |
| --- | --- | --- | --- | --- | --- | --- | --- |
| 1 | Sennoside C | [C_42_H_40_O_19_](https://pubchem.ncbi.nlm.nih.gov/#query=C42H40O19) | 37271-16-2 | 31 | Chebulagic acid | C_41_H_30_O_27_ | 23094-71-5 |
| 2 | Sennoside E_qt | C_55_H_38_O_23_ | 11137-63-6 | 32 | Chebulinic acid | C_41_H_32_O_27_ | 18942-26-2 |
| 3 | Ellagic acid | C_14_H_6_O_8_ | 476-66-4 | 33 | Chebupentol | C_30_H_50_O_5_ | 143086-38-8 |
| 4 | Benzoic acid | C_7_H_6_O_2_ | 65-85-0 | 34 | Punicalagin | C_48_H_28_O_30_ | 65995-63-3 |
| 5 | Linoleic acid | C_18_H_32_O_2_ | 60-33-3 | 35 | (R)-(6-methoxy-4-quinolyl)-[(2R,4R,5S)-5-vinylquinuclidin-2-yl]methanol | C_20_H_24_N_2_O_2_ | 130-95-0 |
| 6 | 1-O-Galloyl-glycerol | C_10_H_12_O_7_ | 59634-75-2 | 36 | apigenin | C_15_H_10_O_5_ | 520-36-5 |
| 7 | Butylated hydroxytoluene | C_15_H_24_O | 128-37-0 | 37 | 2,6-Dimethylheptadecane | C_19_H_40_ | 54105-67-8 |
| 8 | Quinic acid | [C_7_H_12_O_6_](https://pubchem.ncbi.nlm.nih.gov/#query=C7H12O6) | 77-95-2 | 38 | 1,6-bis-O-galloyl-β-D-glucose | C_20_H_20_O_14_ | - |
| 9 | Ethyl geranate | C_12_H_20_O_2_ | 13058-12-3 | 39 | Catharanthamine | C_46_H_56_N_4_O_9_ | 78779-58-5 |
| 10 | Shikimic acid | C_7_H_10_O_5_ | 138-59-0 | 40 | Cheilanthifoline | C_19_H_19_NO_4_ | 483-44-3 |
| 11 | Corilagin | C_27_H_22_O_18_ | 23094-69-1 | 41 | Terchebin | C_41_H_30_O_27_ | 20598-45-2 |
| 12 | Gallic acid | C_7_H_6_O_5_ | 149-91-7 | 42 | Neochebulagic acid | C_41_H_32_O_28_ | 28196-46-5 |
| 13 | Melissic acid | C_30_H_60_O_2_ | 506-50-3 | 43 | Methyl gallate | C_8_H_8_O_5_ | 99-24-1 |
| 14 | Corosolic acid | C_30_H_48_O_4_ | 4547-24-4 | 44 | Terchebulin | C_48_H_28_O_30_ | 132854-40-1 |
| 15 | Pentadecane | C_15_H_32_ | 629-62-9 | 45 | Arjungenin | C_30_H_48_O_6_ | 58880-25-4 |
| 16 | 7-Dehydrosigmasterol | C_29_H_50_O | - | 46 | Daucosterol | C_35_H_60_O_6_ | 474-58-8 |
| 17 | Gallic acid-3-O-(6'-O-galloyl)glucoside | C_20_H_20_O_14_ | - | 47 | Terminolic acid | C_30_H_48_O_6_ | 564-13-6 |
| 18 | Chebulic acid | C_14_H_12_O_11_ | 23725-05-5 | 48 | β-sitosterol | C_29_H_50_O | 83-46-5 |
| 19 | Palmitic acid | C_16_H_32_O_2_ | 1957-10-3 | 49 | Chebuloside-Ⅱ | C_36_H_58_O_11_ | 149475-28-5 |
| 20 | Arjunolic acid | C_30_H_48_O_5_ | 465-00-9 | 50 | Maslinic acid | C_30_H_48_O_4_ | 4373-41-5 |
| 21 | Linoelaidic acid | C_18_H_32_O_2_ | 2197-37-7 | 51 | Quercetin dihydrate | C_15_H_14_O_9_ | 6151-25-3 |
| 22 | α-Santalol | C_15_H_24_O | 115-71-9 | 52 | Quercetin-3-L-rhamnoside | C_21_H_20_O_11_ | 522-12-3 |
| 23 | Pentagalloylglucose | C_42_H_32_O_26_ | 14937-32-7 | 53 | (2Z)-2-[(E)-3-cyano-3-[(2S,3R,4S,5S,6R)-3,4,5-trihydroxy-6-(hydroxymethyl)oxan-2-yl]oxyprop-2-enylidene]butanedioic acid | C_14_H_17_NO_10_ | - |
| 24 | 3-Dehydroshikimate | C_7_H_8_O_5_ | 2922-42-1 | 54 | L-aspartic acid | C_4_H_7_NO_4_ | 56-84-8 |
| 25 | D-Altro-3-heptulose | C_7_H_14_O_7_ | 13059-96-6 | 55 | L-glutamic acid | C_5_H_9_NO_4_ | 56-86-0 |
| 26 | Ellipticine | C_17_H_14_N_2_ | 519-23-3 | 56 | 1,3,6-Tri-O-galloyl-beta-D-glucose | C_27_H_24_O_18_ | 18483-17-5 |
| 27 | Peraksine | C_19_H_22_N_2_O_2_ | 15527-80-7 | 57 | L-lysine | C_6_H_14_N_2_O_2_ | 56-87-1 |
| 28 | Teresautalic acid | C_10_H_14_O_2_ | - | 58 | L-proline | C_5_H_9_NO_2_ | 147-85-3 |
| 29 | Triglochinin_qt | C_14_H_17_NO_10_ | 28876-11-1 | 59 | Hexadecane-d34 | C_16_H_34_ | 15716-08-2 |
| 30 | Beta-Glucogallin | C_13_H_16_O_10_ | 58511-73-2 | 60 | L-arginine | C_6_H_14_N_4_O_2_ | 74-79-3 |

**Table S2.** TRPV1 antagonists and TRPV1 agonists.

| NO. | TRPV1 Antagonist | Renference | NO. | TRPV1 Agonist | Renference |
| --- | --- | --- | --- | --- | --- |
| 1 | MK-2295 | (Li et al., 2011) | 1 | Resiniferatoxin (RTX) | (Gunthorpe and Szallasi, 2008) |
| 2 | AMG-517 | (Burgess and Williams, 2010) | 2 | Compound 3 | (Duarte et al., 2020) |
| 3 | SB-705498 | (Burgess and Williams, 2010) | 3 | DA-5018 | (Li et al., 2011) |
| 4 | K-685 | (Sugimoto et al., 2013) | 4 | Compound 2 | (Duarte et al., 2020) |
| 5 | ABT-102 | (Li et al., 2011) | 5 | Piperine | (Szolcsányi and Sándor, 2012) |
| 6 | Capsazepine | (Gavva, 2008) | 6 | Compound 1 | (Duarte et al., 2020) |
| 7 | BCTC | (Nie, 2020) | 7 | Capsaicin | (Szolcsányi and Sándor, 2012) |
| 8 | PAC-14028 | (Choi et al., 2020) | 8 | 6-Gingerol | (Yin, 2019) |
| 9 | SB-366791 | (Mazeto et al., 2020) | 9 | Zingerone | (Yin, 2019) |
| 10 | SC-0030 | (More et al., 2020) | 10 | 6-Shogaol | (Yin, 2019) |

**Table S3.** The TRPV1 agonists with the same EC_50_ data source and their pEC_50_ values.

| NO. | Compound | pEC_50_ | NO. | Compound | pEC_50_ |
| --- | --- | --- | --- | --- | --- |
| 1 | BDBM50247741 | 0.97 | 7 | BDBM50247746 | -0.59 |
| 2 | BDBM50366620 | 0.56 | 8 | BDBM50247749 | -0.62 |
| 3 | BDBM50247743 | -0.25 | 9 | BDBM50247744 | -0.67 |
| 4 | BDBM50247748 | -0.26 | 10 | BDBM50247751 | -0.74 |
| 5 | BDBM50247745 | -0.28 | 11 | BDBM50247747 | -0.96 |
| 6 | BDBM50247742 | -0.39 | 12 | BDBM50247750 | -1.05 |

**Table S4.** The results of all compounds in CF docking with 5IRX, 5IS0.

| NO. | Compound | Scores(5IRX) | Scores(5IS0) |
| --- | --- | --- | --- |
| 1 | Apigenin | -7.3 | -7.0 |
| 2 | (2Z)-2-[(E)-3-cyano-3-[(2S,3R,4S,5S,6R)-3,4,5-trihydroxy-6-(hydroxymethyl)oxan-2-yl]oxyprop-2-enylidene]butanedioic acid | -6.8 | -6.0 |
| 3 | 1,3,6-Tri-O-galloyl-*β*-D-glucoside | -7.8 | -8.5 |
| 4 | 1,6-bis-O-galloyl-*β*-D-glucose | -7.9 | -7.5 |
| 5 | 1-O-Galloyl-glycerol | -6.5 | -6.8 |
| 6 | 2,6-Dimethylheptadecane | -5.7 | -4.9 |
| 7 | 3-Dehydroshikimate | -5.5 | -5.7 |
| 8 | 7-Dehydrosigmasterol | -8.2 | -8.0 |
| 9 | Arjungenin | -7.7 | -7.0 |
| 10 | Sennoside C | -6.8 | -7.6 |
| 11 | Arjunolic acid | -7.4 | -7.0 |
| 12 | Benzoic acid | -5.4 | -5.7 |
| 13 | *β*-Glucogallin | -7.5 | -6.9 |
| 14 | butylated hydroxytoluene | -5.9 | -5.3 |
| 15 | Catharanthamine | -8.2 | -7.2 |
| 16 | Chebulagic acid | -7.7 | -6.4 |
| 17 | Chebulic acid | -5.8 | -6.3 |
| 18 | Chebulinic acid | -7.8 | -7.7 |
| 19 | (R)-(6-methoxy-4-quinolyl)-[(2R,4R,5S)-5-vinylquinuclidin-2-yl]methanol | -6.4 | -6.3 |
| 20 | Chebuloside-Ⅱ | -8.4 | -7.1 |
| 21 | Chebupentol | -7.5 | -6.4 |
| 22 | Cheilanthifoline | -7.5 | -7.9 |
| 23 | Corilagin | -7.0 | -7.9 |
| 24 | Corosolic acid | -8.5 | -7.4 |
| 25 | D-Altro-3-heptulose | -5.1 | -6.5 |
| 26 | Daucosterol | -8.1 | -9.1 |
| 27 | Ellagic acid | -6.7 | -7.5 |
| 28 | Ellipticine | -7.0 | -9.2 |
| 29 | Ethyl geranate | -6.0 | -6.1 |
| 30 | Gallic acid | -6.4 | -6.3 |
| 31 | Gallic acid-3-O-(6'-O-galloyl)glucoside | -8.4 | -7.8 |
| 32 | Hexadecane-d34 | -4.8 | -5.3 |
| 33 | L-arginine | -5.5 | -6.5 |
| 34 | L-aspartic acid | -5.0 | -5.4 |
| 35 | Linoelaidic acid | -6.0 | -6.6 |
| 36 | Linoleic acid | -6.0 | -6.7 |
| 37 | L-lysine | -5.0 | -5.6 |
| 38 | L-proline | -5.0 | -5.3 |
| 39 | Maslinic acid | -8.6 | -7.5 |
| 40 | Melissic acid | -4.9 | -5.6 |
| 41 | Methyl gallate | -6.6 | -6.5 |
| 42 | Neochebulagic acid | -7.1 | -7.2 |
| 43 | Palmitic acid | -6.1 | -6.1 |
| 44 | Pentadecane | -4.6 | -4.9 |
| 45 | Pentagalloylglucose | -6.5 | -6.0 |
| 46 | Peraksine | -7.4 | -6.8 |
| 47 | Punicalagin | -5.5 | -0.8 |
| 48 | Quercetin dihydrate | -1.3 | -1.7 |
| 49 | Quercetin-3-L-rhamnoside | -8.2 | -7.1 |
| 50 | Quinic acid | -5.9 | -5.7 |
| 51 | Sennoside C | -6.8 | -7.6 |
| 52 | Sennoside E_qt | -7.3 | -7.3 |
| 53 | Shikimic acid | -6.0 | -5.9 |
| 54 | Terchebin | -7.8 | -8.3 |
| 55 | Terchebulin | -7.7 | -0.9 |
| 56 | Teresautalic acid | -5.0 | -5.3 |
| 57 | Terminolic acid | -8.1 | -7.1 |
| 58 | Triglochinin_qt | -6.0 | -6.2 |
| 59 | α-Santalol | -6.0 | -6.3 |
| 60 | β-sitosterol | -7.5 | -8.1 |

**
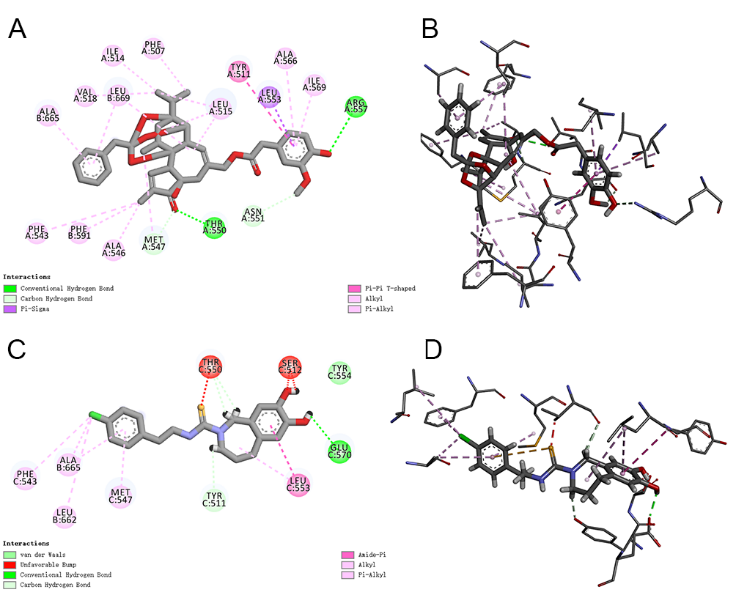
**

**Figure S1.** Diagram of interaction between the protein–ligand complex. (A) The 2D binding mode of the 5IRX and RTX. (B) The 3D binding mode of the 5IRX and RTX. (C) The 2D binding of the 5IS0 and capsazepine. (D) The 3D binding of the 5IS0 and capsazepine.
